# Reclaiming urban space for children: Examining the impact of School Streets on independent mobility and active transport

**DOI:** 10.17269/s41997-025-01058-w

**Published:** 2025-06-09

**Authors:** Zeinab Aliyas, Carise Thompson, Fanny Bakkali, Patricia A. Collins, Katherine L. Frohlich

**Affiliations:** 1 Centre de Recherche en Santé Publique (CReSP), Université de Montréal, Montreal, Québec Canada; 2 Department of Geography and Planning, Queen’s University, Kingston, Ontario Canada; 3 École de Santé Publique, Université de Montréal, Montreal, Québec Canada

**Keywords:** School Street, Active transportation, Children’s modal shift, Children’s independent mobility, Rue-école, Transport actif, Le transfert modal des enfants, La mobilité indépendante des enfants

## Abstract

**Intervention:**

School Street initiatives are traffic-free zones around the entrances of primary schools designed to make it safer for children to come and go from school. Beyond increasing safety, these initiatives offer opportunities for children to increase their engagement in active school travel and to become more independently mobile.

**Research question:**

How does the implementation of a School Street intervention influence children’s active travel and independent mobility on their way to school?

**Methods:**

A School Street intervention was implemented every Friday at a primary school in Montreal from September 2022 to June 2023. Bi-monthly direct observations were conducted to collect data on children’s modes of travel and whether they travelled independently or with an adult before the start of classes.

**Results:**

Independent, active travel was more frequently observed on School Street days compared to non-School Street days. Additionally, a progressive increase in active transportation and independent mobility was observed throughout the school year, regardless of School Street designation.

**Conclusion:**

This study underscores the potential impact of School Street interventions on enhancing children’s active school travel and independent mobility. Establishing safe zones near schools can positively influence children’s commuting behaviours, fostering healthier and more autonomous travel habits.

## Résumé

**Intervention:**

Les rues-écoles sont des zones sans circulation qui sont situées aux abords des écoles primaires, conçues pour rendre le trajet des enfants plus sûrs entre l’école et leur domicile. Au-delà de l’amélioration de la sécurité, ces initiatives offrent aux enfants la possibilité de s’engager davantage dans des déplacements scolaires actifs et de devenir plus autonomes dans leur mobilité.

**Question de recherche:**

Comment la mise en œuvre d’une intervention de rue-école influence-t-elle les déplacements actifs et la mobilité indépendante des enfants sur leur trajet pour se rendre à l’école?

**Méthodes:**

De septembre 2022 à juin 2023, une intervention de rue-école a été mise en œuvre tous les vendredis dans une école primaire de Montréal. Des observations directes bimensuelles ont été effectuées pour recueillir des données sur les modes de déplacement des enfants et pour comprendre s’ils se déplaçaient de manière indépendante ou avec un adulte pour se rendre à l’école le matin.

**Résultats:**

Les déplacements indépendants et actifs étaient plus fréquemment observés les jours de rues-écoles comparativement aux journées sans rues-écoles. En outre, une augmentation progressive du transport actif et de la mobilité indépendante a été observée tout au long de l’année scolaire, même les journées sans rues-écoles.

**Conclusion:**

Cette étude souligne l’impact potentiel des interventions de rues-écoles sur l’amélioration du transport scolaire actif et sur la mobilité indépendante des enfants. Établir des zones de sécurité près des écoles peut positivement influencer les comportements de déplacement des enfants, favorisant des habitudes de déplacement plus saines et autonomes.

## Introduction

Urban children’s engagement in active and independent travel to school, such as walking, biking, or rolling, has been in decline in recent decades in countries around the world (Aranda-Balboa et al., 2020; Bhosale et al., 2017; Reimers et al., 2021). Aranda-Balboa et al. (2020) observed a decrease in children’s active school travel (AST) between 2001 and 2011 in North America, with a reduction of 8% in Canada and 20% in the United States. Another recent study reported that, as of 2021, nearly half of children still rely on motor vehicle transportation to commute to school (Rothman et al., 2021). This high use of motorized transport exists despite the well-established benefits of AST to children’s physical, mental, and social health (Stark et al., 2018; Waygood et al., 2017).

Scholars believe that these changing mobility patterns are likely the consequence of a mix of unsupportive built and social environments, as well as individual- and household-level factors (Chaput et al., 2020). Some of these factors are modifiable (e.g., parent and child perceptions), while others are not (e.g., age, ethnicity). Among the modifiable factors impacting transport choices, perceived danger is a primary constraint on children’s independent and active mobility (Marzi et al., 2018). Specifically, children of parents who express greater safety concerns tend to travel actively and independently to school less often than those of parents not voicing these concerns (Lee et al., 2023; Marzi et al., 2018). Similarly, there is some evidence to show that children’s perceptions about safety, including perceptions about traffic and crime, may also constrain their independent active school travel (Lee et al., 2023).

Canadian scholars have identified several modifiable factors within the built environment that may be constraining AST and independent mobility (IM)—the ability for children to freely travel around their neighbourhood or city without adult supervision. For instance, built environments prioritizing automobiles over human-scale considerations have led to increased traffic volumes around schools. Research has shown that more vehicular traffic around schools decreases the odds of a child walking independently (Buliung et al., 2017). On the other hand, the presence of school crossing guards and cycling infrastructure, as well as areas achieving a higher Walk Score® (indicating greater walkability) and density of traffic signals, have been associated with increased odds of active school travel in Canadian cities (Rothman et al., 2021). Additionally, recognizing that the design of urban areas influences not only traffic flow but also pedestrian safety and accessibility is important. Cities characterized by faster speed limits, narrow sidewalks, and a lack of speed bumps may inadvertently discourage walking and cycling. Moreover, the proliferation of automobiles within cities can further exacerbate these challenges, directly impacting children’s active school travel (Wong et al., 2011). Thus, declining rates of children’s AST and IM may be the result of the evolution of urban environmental design which has not prioritized children’s needs to move freely in their communities. As a result, children are increasingly dependent on their parents to transport them to and from school, even those living within 5 min of their school (Lavergne et al., 2023).

As a result of these trends, interventions that aim to increase rates of AST have grown in popularity over the past decade (Public Health Agency of Canada, 2023). Some of the most common AST interventions include safe routes to school programs, school travel plans, walking school buses, and curriculum-based interventions (Larouche et al., 2018). However, the evidence is inconclusive regarding the effectiveness of AST interventions in influencing modal shift (Larouche et al., 2018). Oftentimes AST interventions employ more than one approach (i.e., more than one of the “6 E’s”: engagement, equity, engineering, encouragement, education, and evaluation), which makes evaluating the distinct outcomes difficult (Buttazzoni et al., 2018). However, Buttazoni et al. (2018) suggest that AST interventions with longer-term follow-up periods (i.e., > 6 months) may be more effective at changing the prevalence of AST compared to those with shorter-term follow-ups (i.e., 6 months or less). This implies the importance of implementing and evaluating interventions over more extended periods to study and potentially achieve significant changes.

Emerging evidence reveals that the concept of children’s IM should not be left out of this conversation, as IM is closely, and positively, tied with AST (Larouche et al., 2020). Both AST and IM are associated with health-promoting outcomes for children, including co-benefits like increased time spent in moderate-to-vigorous physical activity, enhanced social competencies, cognitive development, spatial awareness, and psychosocial well-being (Webb Jamme et al., 2018). These outcomes arise not only because children are exercising and often socializing when engaged in AT, but also because, when actively commuting to school independently of their parents or caregivers, children must make decisions and solve problems without parental guidance in order to navigate traffic, their environment, and unforeseen obstacles. Compared to children who are driven to school, active commuters demonstrate enhanced spatial knowledge of their environment and sense of community (Webb Jamme et al., 2018). Additional evidence shows that children who walk, cycle, or use other active means on their school journey tend to perform better academically (Singh et al., 2012). Beyond the short term, travelling behaviours formed in youth are important for establishing lifelong behaviours and thus have significant implications for population health (Dimitri et al., 2020). Making progress on these varied health outcomes aligns with Keeley (2021) and Levy et al. (2020) in support of children’s population health.

School Street interventions involve a multi-pronged approach to increasing AST and IM by simultaneously addressing objective hazards posed by motor vehicles around schools and the subjective perceptions held by parents and children that limit engagement in AST and IM. School Streets are designated roadways outside primary school entrances that are temporarily closed to motorized traffic during drop-off and pick-up times during the school day. By drastically reducing or eliminating motor vehicle traffic adjacent to schools, School Streets create a safe zone for children coming and going from school by active modes of travel in areas that were once inundated by vehicular congestion. Implemented in cities worldwide such as London, New York, and Paris, School Streets have also been introduced in several Canadian cities in recent years, including Vancouver, Winnipeg, Kingston, Mississauga, and Montreal.

Despite the growing popularity of School Streets, little is known about whether such interventions increase active trips to school (8 80 Cities, 2022; IVA Mobiliteitsbredijf Stad Gent, 2017) or foster greater independent mobility among children (Lee et al., 2021). These research gaps highlight the need for more extensive and targeted studies to comprehend the multifaceted effects of School Streets on children’s travel behaviours and their IM. In this regard, this study aims to (1) compare children’s modes of travel to school on School Street days vs non-School Street days and (2) compare rates of children’s independent travel to school on School Street days vs non-School Street days, during a full school year.

## Methods

### Study setting

This study is part of a larger research program, which evaluated the implementation and outcomes associated with School and Play Street pilot interventions in Montreal (Quebec) and Kingston (Ontario). The purpose of these interventions was to create the conditions for children to safely engage in active school travel and outdoor free play independently of their parents and caregivers.

The findings presented in this paper are derived from data collected during a School Street initiative implemented at a primary school in a borough in the north-east of Montreal. This borough is large and diverse, with significant socioeconomic contrasts, where some areas are affluent, while others face higher levels of economic hardship. The borough is home to approximately 118,000 residents. The district is also highly diverse, with a large proportion of residents born outside Canada. While it includes long-established communities, it has also seen significant recent immigration, making it one of the boroughs with the highest number of newcomers in the city. The full details of the city, borough, and district in which this School Street is located are presented in Table 1.

**Table 1:** Sociodemographic characteristics of the School Street’s location at the city, borough, and district levels (2021 Census data)

	City of Montreal, Quebec (Census subdivision)	Borough	District
Population (2021)	1,762,949	118,170	10,984
Total—knowledge of official languages for the total population excluding institutional residents—100% data			
English and French	58%	59%	53%
French only	27%	30%	30%
English only	12%	8%	12%
Neither English nor French	2%	3%	5%
Distribution (%) of the population by broad age groups—100% data			
0 to 14 years	15%	17%	18%
15 to 64 years	68%	64%	68%
65 years and over	17%	19%	14%
Average age of the population	40.6	41.7	38.6
Median age of the population	38.8	40.8	37.2
Immigrant status for the population in private households—25% sample data			
Non-immigrants	59%	56%	49%
Immigrants	33%	39%	41%
Non-permanent residents	8%	5%	10%
Income status			
Median total income of household in 2020 ($)	$63,600.00	$65,000.00	$59,200.00
Median after-tax income of household in 2020 ($)	$56,000.00	$56,800.00	$52,800.00

The school is located in a district within the borough with a higher proportion of lower-income households compared to the citywide average. The School Street site is located on a residential street, surrounded by a grid-like street pattern. This area is busier than the adjacent streets because it is next to one of the borough’s main shopping streets. It is also frequently used as a shortcut to major roads leading to the freeway.

The selected primary school has approximately 355 students, offering regular programs from kindergarten (5 years of age) to 6th grade (12 years of age). The school has two entrances: the main entrance, located on the street where the School Street intervention was implemented, and a secondary entrance on another street, which connects to a public open space adjacent to the school. This open space includes various play facilities, with sand-covered ground in the play area, water games available in warmer seasons, a synthetic soccer pitch, additional grass-covered playing fields, and a basketball court. The park also features a canopy of mature and young trees, providing shade along pathways and at the park’s edges. Sidewalks are present throughout the school area. Intersection signage is limited to stop signs, which are not always respected by motorists. Crosswalks exist but are not consistently well marked. There are no traffic lights, pedestrian beg buttons, or button-controlled crosswalks, and road markings for pedestrian crossings are minimal, often limited to faded painted lines rather than zebra-striped crossings. Speed limit signs are present at both entrances to the School Street, reinforcing the reduced speed zone. The total distance of the School Street section is 200 m.

The School Street operations are managed by three people for each shift: two individuals are stationed at each entrance of the School Street, while one person is positioned within the street itself to guide local vehicles entering or exiting the closed zone. The deployment of temporary lightweight barricades allowed for exempted motorists (e.g., school shuttle services and buses) to enter and exit the closed zone as needed. In practice, school shuttle services were the primary vehicles entering and exiting the zone (children who travelled this way were not counted), along with a few residents who exited the zone by personal vehicle during the School Street sessions.

The School Street sessions were held every Friday over a period of the school year. Morning sessions commenced 40 min before the school day began, and afternoon sessions ran for 55 min after the school day. Observations were limited to the morning sessions since a high proportion of the school’s student body engaged in after-school programs and thus did not leave the site at the school’s formal dismissal time. Conducting morning observations thus improved the validity of the data, as all children could be included in the travel mode counts.

### Measures

#### Active transport and independent mobility

An adapted version of the child pedestrian injury and active school transportation toolkit was utilized to evaluate children’s AST and IM (Toronto & Peel, 2018). In the original version, the assessment only considered whether children travelled by car, foot, or bicycle/scooter. In the adapted version, the evaluation of active travel was extended to encompass independence, including using AT alone, in groups with other children, or accompanied by adults. This expanded approach provides a more comprehensive understanding of children’s mobility patterns during their journey to school. Therefore, children were classified into two main groups based on their mode of travel to school: active travelers (those who walked, cycled, or used a scooter) and passive travelers (those who travelled by car). Among active travelers, we further distinguished between independent travel—children walking or cycling alone or with peers only—and accompanied travel, where children actively travelled and were accompanied by an adult, regardless of whether other children were also present. Accordingly, only children in the independent travel group were included under IM, while both independent and accompanied active travellers were included under AST.

### Observation method

**Fig. 1:**
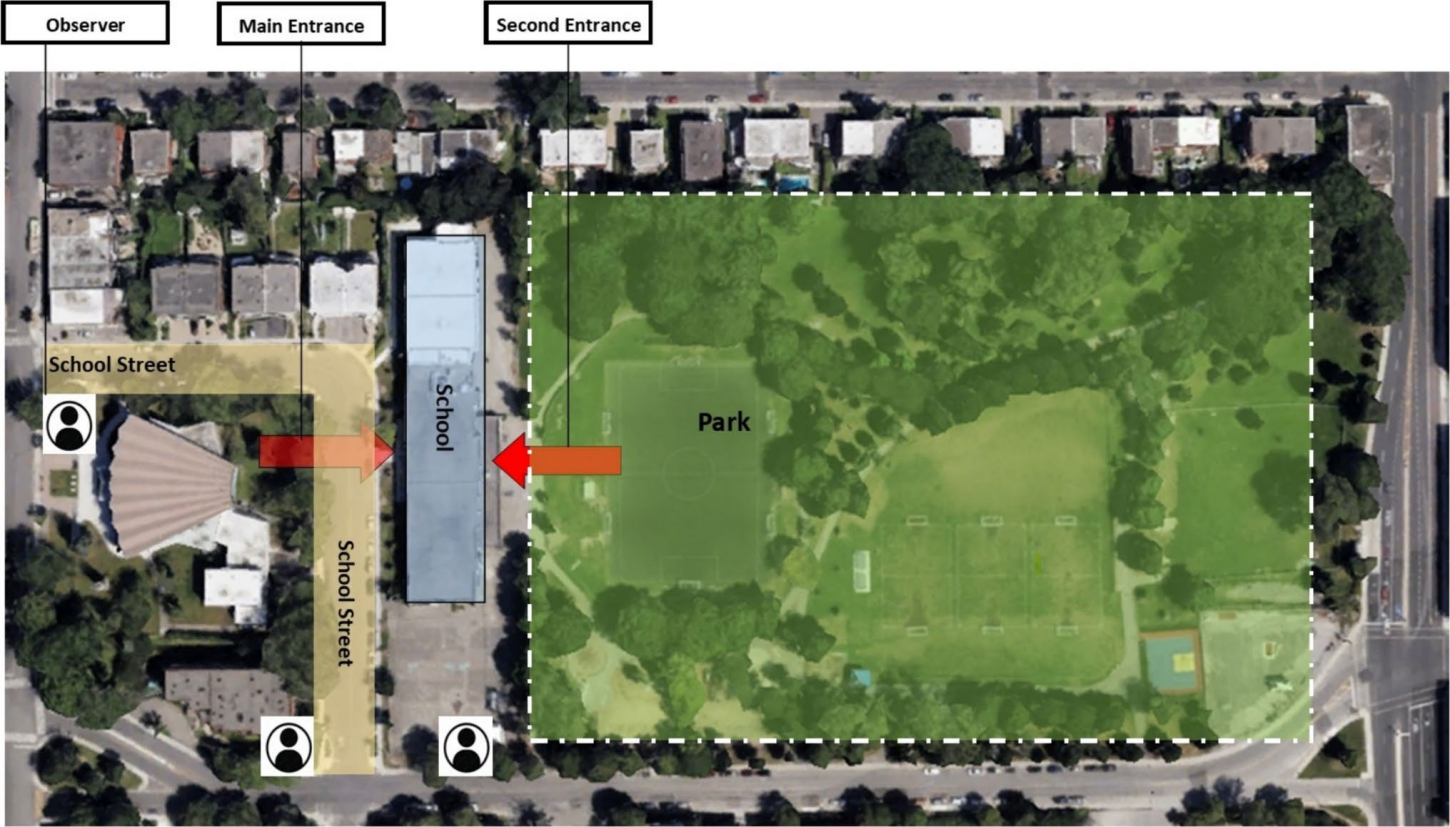
Map of observation points

Trained research assistants made observations twice monthly, including during one School Street day (i.e., Friday) and one non-School Street day (i.e., any day from Monday to Thursday). Each set of observations was taken during the 40 min before school began. Both observation sessions occurred within the same week, with the non-School Street day randomly selected each month to capture data across various weekdays. Additionally, during each observation, weather conditions and temperature were noted. To ensure reliability, all observers underwent standardized, on-site training before data collection, including a pilot test to assess inter-rater reliability, which ranged from 0.77 to 0.91—indicating acceptable agreement (Landis & Koch, 1977). During each observation session, three observers were deployed: two at opposite sides of the School Street’s busier entrance and one at a quieter entrance outside the School Street zone (Fig. 1). While resource constraints prevented the use of double observers at each station, observer consistency was maintained by assigning individuals to the same locations throughout the study. The research assistants arrived 5 min before the observation period began to enable assessments of the environment around the school, such as temperature and road conditions (dry, slippery, icy). Observers systematically recorded all observations using a standardized form that was validated and tested in the pilot phase at the beginning of the intervention, categorizing the number of children passing by them according to the specified criteria outlined in the form. Summaries were made each day, totaling the number of students observed using the various transportation modes at each observation location.

### Data analysis

All data were analyzed utilizing both Excel and SPSS version 25. Descriptive statistics were generated and visually depicted in graphs to represent the diverse patterns observed in AST and IM across each month. Additionally, changes in AST and IM from School Street days to non-School Street days were presented as percentages to illustrate differences in children’s active mobility across different months. To compare AST and IM between School Street days and non-School Street days, we first presented the total number of observed children as 100%, then extracted the number and percentage of those who actively travelled to school (either AST or IM in Tables 1 and 2). Differences between School Street days and non-School Street days were expressed as percentages to highlight variations in children’s active mobility across months. To calculate the relative percentage increase in IM and AST on School Street days compared to non-School Street days, we used the following formula: (School Street day percentage − Non-School Street day percentage)/Non-School Street day percentage × 100. Additionally, mixed-effects models, employing linear regressions, were also developed to investigate the potential association of AST and IM with School Street and non-School Street days.

**Table 2:** Comparison of children engaged in AST on School Street days vs non-School Street days across months

Months	Weather on School Street days	Total observed children on School Street days	AST on School Street days	Weather on non-School Street days	Total observed children on non-School Street days	AST on non-School Street days	Difference in AST on School Street days vs non-School Street days
	(°C)	***n***	***n*** (%)	(°C)	***n***	***n***(%)	%
Sept	Cloudy (6)	200	173 (87%)	Cloudy (11)	232	162 (70%)	24
Oct	Cloudy (14)	223	192 (86%)	Sunny (4)	210	155 (74%)	16
Nov	Sunny (8)	223	190 (85%)	Sunny (2)	215	151 (70%)	21
Dec	Sunny (− 4)	215	180 (84%)	Rainy (6)	214	154 (72%)	17
Jan	Snowy (− 2)	219	189 (86%)	Sunny (− 15)	196	167 (85%)	1
Feb	Snowy (− 12)	211	171 (81%)	Sunny (− 2)	225	161 (72%)	13
March	Sunny (− 3)	218	178 (82%)	Sunny (− 4)	195	155 (79%)	4
April	Sunny (6)	201	153 (76%)	Sunny (4)	200	167 (84%)	− 10
May	Sunny (16)	194	170 (88%)	Sunny (12)	206	147 (71%)	24
June	Cloudy (17)	232	191 (82%)	Cloudy (21)	173	150 (87%)	− 6
Average		213	179 (84%)		206	157 (76%)	10

## Results

A maximum of 232 children were observed during 20 observation sessions (10 sessions on School Street days and 10 on non-School Street days), generating a combined total of 4202 data points for children’s travel behaviour to school. Since the school has 355 students, the same children were observed repeatedly across sessions, explaining the total data points. While some children were counted multiple times on different days, others may have varied between sessions.

### Children’s active transportation to school

**Fig. 2:**
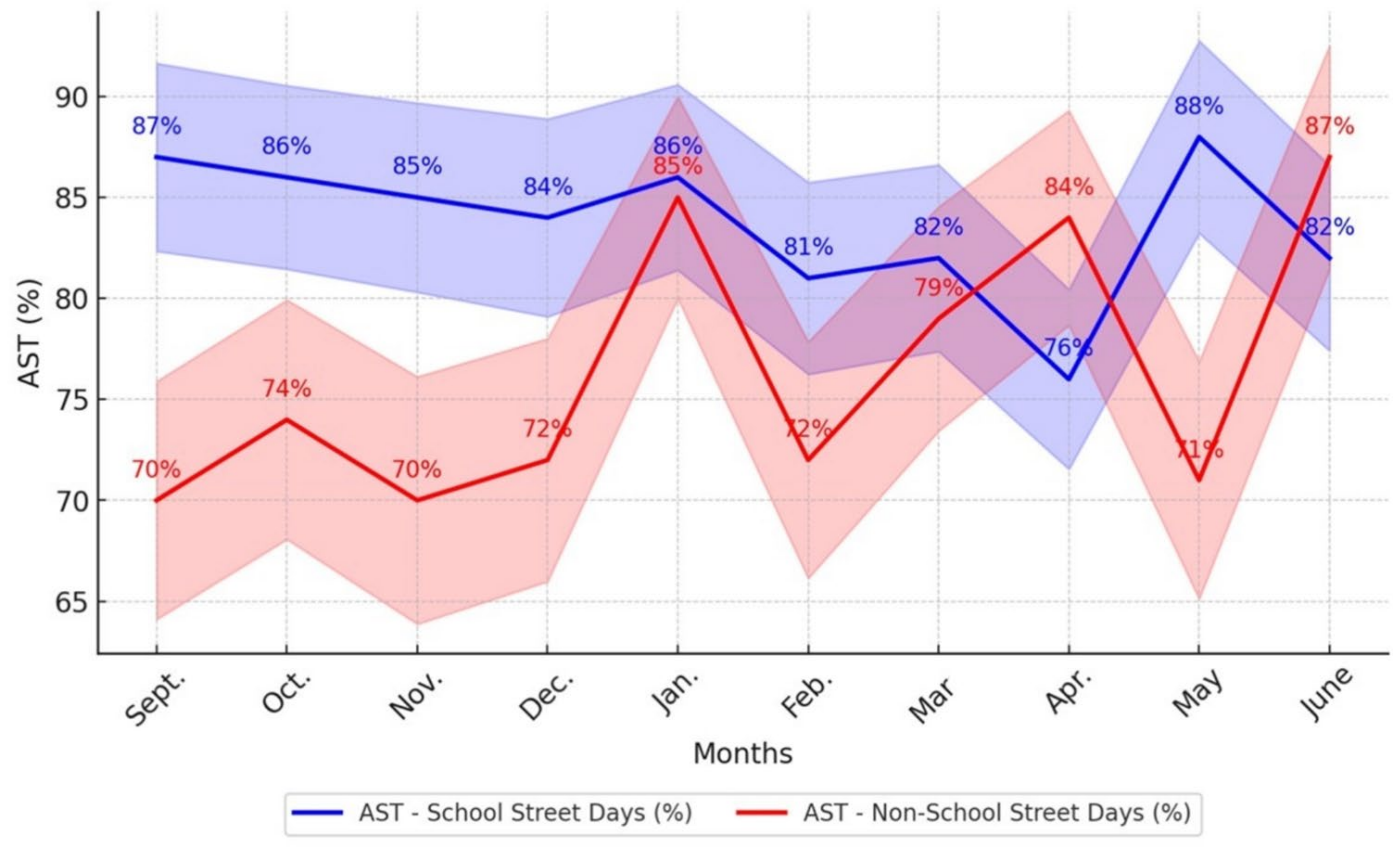
Monthly variation in AT (%) on School Street days vs non-School Street days

Across the 10 months of observations, the instances of children engaging in AST were consistently higher on School Street days compared to non-School Street days, except for April and June. The smallest differences between School Street and non-School Street days occurred in January, coinciding with colder weather, while the largest disparities in AST were observed in September and May (Table 2). The findings revealed a consistently high rate of AST among children every month, both on School Street days and non-School Street days. Throughout the 2022–2023 school year, over 70% of observed students at the school engaged in AST. Notably, AST rates remained steadily high (between 70% and 86%) across all observations, suggesting that seasonal changes and weather conditions had minimal impact on children’s AST behaviours (Fig. 2).

### Children’s independent mobility

**Fig. 3:**
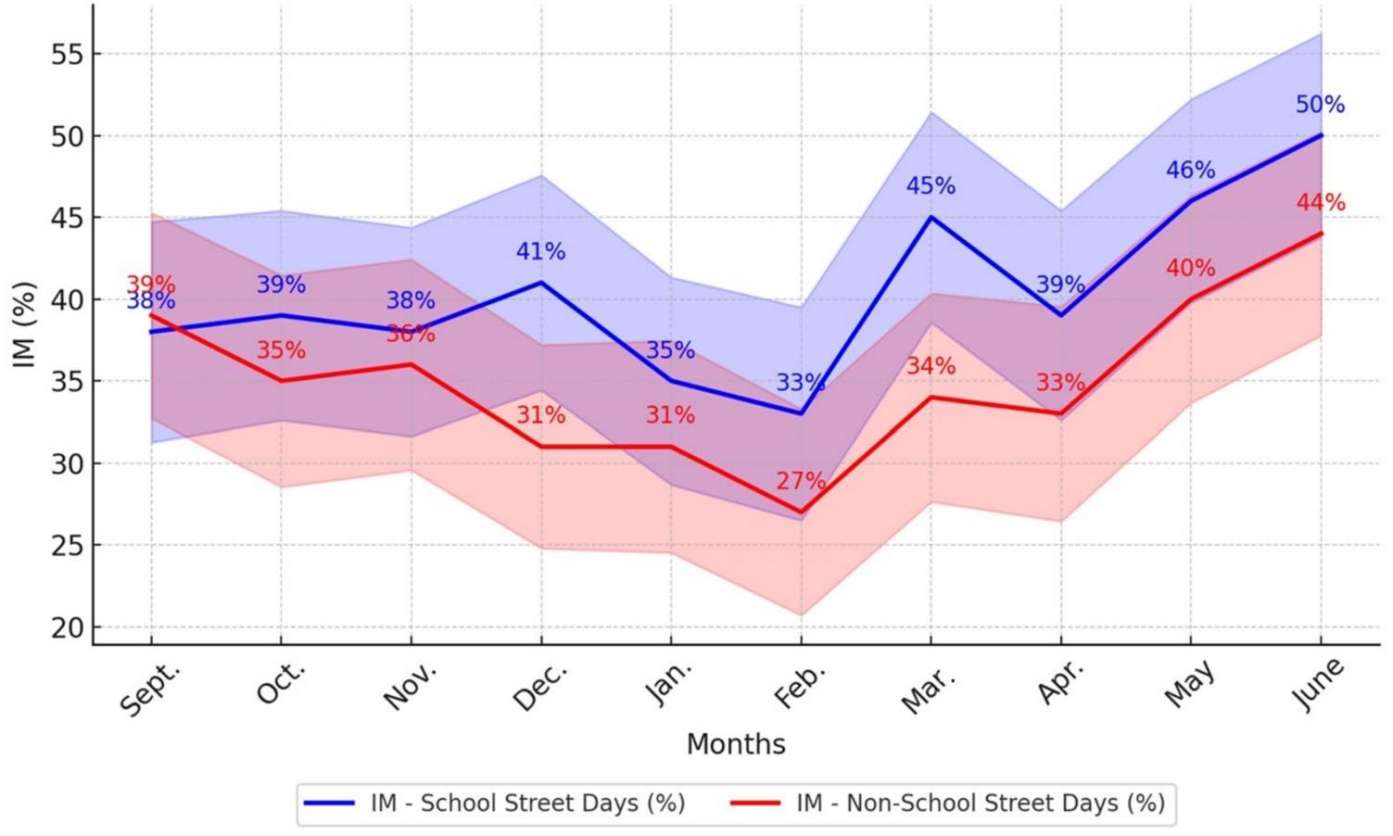
Monthly variation in IM (%) on School Street days vs non-School Street days

Across the 10 months of observation, a higher incidence of IM on School Street days was observed compared to non-School Street days, except during September (− 3%), the initial month of the intervention. Comparing results between School Street and non-School Street days revealed that the largest disparity was observed in December and March, with 32% more children engaging in IM on School Street days. The smallest difference occurred in September, with fewer children participating in IM on School Street days than on non-School Street days (Table 3 and Fig. 3).

**Table 3:** Comparison of children engaging in IM on School Street days vs non-School Street days across months

Months	Weather on School Street days	Total observed children on School Street days	IM on School Street days	Weather on non-School Street days	Total observed children on non-School Street days	IM on non-School Street days	Difference in IM on School Street days vs non-School Street days
	Weather	***n***	***n*** (%)	(°C)	***n***	***n*** (%)	%
Sept	Cloudy (6)	200	76 (38%)	Cloudy (11)	232	90 (39%)	− 3
Oct	Cloudy (14)	223	87 (39%)	Sunny (4)	210	73 (35%)	11
Nov	Sunny (8)	223	85 (38%)	Sunny (2)	215	77 (36%)	6
Dec	Sunny (− 4)	215	89 (41%)	Rainy (6)	214	66 (31%)	32
Jan	Snowy (− 2)	219	76 (35%)	Sunny (− 15)	196	61 (31%)	13
Feb	Snowy (− 12)	211	70 (33%)	Sunny (− 2)	225	61 (27%)	22
March	Sunny (− 3)	218	98 (45%)	Sunny (− 4)	195	66 (34%)	32
April	Sunny (6)	201	78 (39%)	Sunny (4)	200	66 (33%)	18
May	Sunny (16)	194	90 (46%)	Sunny (12)	206	83 (40%)	15
June	Cloudy (17)	232	117 (50%)	Cloudy (21)	173	76 (44%)	14
Average		213	86 (41%)		206	72 (35%)	16

Furthermore, we examined whether there was a statistically significant association between AST and IM on School Street days compared to non-School Street days. The results revealed that AST was 21.8 times more likely to occur (CI, 12.3–31.2; *p* < 0.01), while IM was 14.7 times more likely to occur (CI, 3.6–25.7; *p* < 0.05), on School Street days compared to non-School Street days (Table 4).

**Table 4:** The influence of School Street days on active travel and independent mobility

	Active school travel	Independent mobility
	Unstandardized coefficient (95% CI)	
School Street days	21.8 (12.3–31.2)**	14.7 (3.6–25.7)*

**p < 0.01; *p < 0.05

## Discussion

The findings of this study provide valuable insights into the impacts of School Street interventions on children’s engagement in active and independent modes of travel to school.

First, we observed consistently high rates of AST (over 70%) among children from our study school throughout the year compared to the national average of 47% of children living less than 5 min from school (Public Health Agency of Canada, 2023). This discrepancy suggests a strong inclination towards active modes of travel among this particular student population, regardless of external factors such as weather conditions or seasonal changes, that is considerably different from the norm of student populations in Canadian elementary schools. More research is needed, particularly at schools with more typical rates of baseline AST, to determine how the pre-existing AST culture might influence the effect size of this intervention on AST rates.

This study was conducted in a socioeconomically diverse neighbourhood, where the median income is slightly lower than the citywide average. In this context, parents’ attitudes towards AST may be shaped by a range of factors, including income, education, and work flexibility. While higher socioeconomic status is often associated with greater awareness of AST’s health benefits and the ability to support it—such as through flexible work hours—other elements, like infrastructure, cultural norms, and daily routines, also play important roles. The mixed composition of the neighbourhood suggests that observed changes in AST behaviour likely result from multiple interacting factors beyond socioeconomic status alone, including community engagement, school policies, and the built environment.

Despite the already high baseline rates of AST at this school, the data revealed even higher levels of AST on School Street days compared to non-School Street days, indicating a positive effect of the intervention on AST. Since the School Street intervention ran only on Fridays, however, it is possible that the consistently higher rates of AST on School Street compared to non-School Street days could be attributed to the relative ease of changing one’s mode of travel for a single day of the week. As such, it is unclear whether as much mode shifting would have happened if the intervention ran more frequently, or if it ran on a different day of the week. Thus, future research ought to also examine the effect of frequency and day of the week on AST rates.

Additionally, we observed lower levels of IM in September and AST in April and June on School Street days than on non-School Street days. The lower level of IM in September could be due to the fact that it was the first month of the school year, when parents may have been more likely to accompany their children to help them become familiar with the route and daily routine. Additionally, the School Street intervention had just started in early September, meaning that families were still adjusting to the new traffic changes and were not yet accustomed to using it for active travel. As awareness of the intervention increased and routines stabilized, IM participation may have risen in subsequent months. Regarding April and June, the lower AST levels could be influenced by a combination of seasonal and school-related factors. During these months, students may have participated in off-site activities, school trips, or year-end programs, which could have altered their usual travel routines. Additionally, warmer weather may have encouraged more families to opt for alternative transportation modes, such as driving, due to schedule changes, convenience, or other family obligations. These factors may have disrupted students’ typical use of active school travel during School Street days.

This research enriches the existing literature on the positive impacts of AST interventions on rates of AST (Larouche et al., 2018). While most studies of AST interventions focus on programs (e.g., Walking School Bus), research by McDonald et al. (2014) found that engineering-based interventions (e.g., improvements to sidewalks and bicycle lanes, traffic calming near schools) led to an 18% relative increase in walking and bicycling. School Streets represent a hybrid approach of engineering and programming that may offer more promise for supporting AST than what singular approaches can offer on their own.

Our findings align with numerous reports on School Street pilots from the grey literature (8 80 Cities, 2022; City of Vancouver, 2022; Snaije & Gutierrez, 2022; Transport for London, 2022). These reports found that AST increased from 3% to over 30%, while passive forms of travel (e.g., by private vehicle) decreased during or following the implementation of pilot School Streets (8 80 Cities, 2022; City of Vancouver, 2022; Snaije & Gutierrez, 2022). Comparatively, this study found that the odds of children travelling actively to or from school were 21.8 times greater on School Street days compared to non-School Street days. The increases in AST observed in pilot evaluations of School Streets may be due to increased feelings of safety among children and/or caregivers, reductions in motor vehicle traffic volume and speeds in front of schools, and increased awareness of traffic danger (Transport for London, 2022). Future research could seek to more precisely determine the mechanism(s) for the changes in AST that we, and others, have observed.

Another major finding from this study was the impact of School Streets on children’s IM during their journey to school. The data revealed a consistently higher incidence of IM on School Street days compared to non-School Street days, except for the first month of the intervention. This suggests that the creation of traffic-free zones around school sites through School Street initiatives likely helps to allay parental concerns about safety, which in turn fosters greater independence among children in travelling to school.

Despite a slight decline in IM during the winter months, this study also demonstrated a gradual increase in the number of children engaging in IM throughout the school year, irrespective of whether the School Street was running or not. This finding suggests that prolonged exposure to School Street interventions may positively impact self-efficacy and autonomy among children, and/or perceptions of safety among parents. Additionally, it can be presumed that the presence of the School Street has promoted IM by fostering a positive perception of the social environment among children and improving parent-parent communications, as it created a safe space outside of the school for both children and parents to socialize (Riazi et al., 2021). Alternatively, this trend towards greater independence among the entire student body could reflect increased confidence and maturity that develops with age, and/or decreased protectiveness among parents over the course of the school year. Future School Street evaluations could explore this issue in more detail by including control schools as comparisons.

### Strengths and limitations of the study

Our study benefits from several strengths. First, our data collection spanned an entire school year, allowing us to capture trends and changes in children’s commuting behaviours over time. The study duration of 10 months allowed for more time for behaviour change to occur compared to interventions of shorter durations. This comprehensive approach provides evidence of the sustained effects of School Street interventions on AST and IM. Additionally, our comparative analysis, which included observations on both School and non-School Street days, offers valuable insights into the potential impact of these interventions on children’s travel patterns.

Our study had, however, several limitations that should be acknowledged. Although an established method of observation was used for evaluating travel behaviour (Rothman et al., 2021), this method does not account for mixed modes of transport (i.e., students who drive partway and walk partway). However, the intervention provided a dedicated space for children to walk, covering approximately 200 m, with the school positioned in the middle of the School Street section. This means that all children, regardless of their mode of travel from home, walked at least 100 m from either entrance of the School Street. While our findings do not allow us to determine with complete accuracy whether a child used mixed modes of transport, we know that the intervention resulted in an overall increase in AST for at least some portion of their daily commute. Even if small, this shift contributed to reducing the presence of motorized vehicles, increasing pedestrian activity within the intervention zone, and ultimately enhancing social connection and safety in the area. Additionally, our research was conducted at a single primary school in Montreal, Canada, and as such, our findings are unique to this specific geographical context and cultural setting. The inclusion of a control school would have helped clarify whether the observed increases in AST and IM were solely due to the School Street initiative or if alternative explanations, such as increasing parental comfort or children becoming more familiar with the school route throughout the academic year, could explain these outcomes. Finally, contextual variables that may influence children’s commuting behaviours, such as socioeconomic status and parental attitudes, were not explicitly accounted for in the analysis.

Future research directions should aim to address these limitations and expand upon our findings. For instance, including afternoon sessions and running the intervention for more than one day per week during the observation protocol could provide a more holistic assessment of the impact of School Street interventions on children’s mobility patterns. Moreover, previous research highlights the challenges of evaluating sustained behaviour change and supports AST interventions with longer follow-up periods (i.e., > 6 months) (Buttazzoni et al., 2018). While observations were conducted throughout the 10-month duration of this study, follow-up observations were not conducted in the subsequent school year to effectively measure behaviour change over time. Therefore, future studies should incorporate longer follow-up periods to assess the lasting effects of School Street interventions on children’s travel behaviour. Future studies could benefit from multi-site investigations across diverse urban environments, including those with lower baseline rates of AST, to provide a more comprehensive understanding of the effectiveness of School Street interventions. Finally, collecting data from parents and children regarding the influence of School Street interventions on travel choices would offer contextual insights that cannot be captured through direct observations alone.

## Conclusion

This study contributes to the growing body of evidence supporting the effectiveness of School Street interventions in reclaiming urban space for children and fostering healthier and more sustainable commuting habits. By creating safe, traffic-free environments around school sites, our findings suggest that School Streets have the potential to increase children’s engagement in active school travel and independent mobility. Additionally, increasing children’s independent mobility can provide them with more freedom to walk around their neighbourhoods and a greater opportunity to engage in outdoor activities. In this sense, enhanced freedom can contribute to children’s overall well-being and quality of life, while supporting healthier and more sustainable communities by reducing children’s dependency on motorized forms of transportation. To more fully understand the potential of School Street interventions, future studies should examine their impacts on AST and IM in a diverse range of school settings, at varying levels of exposure, with control groups, and through mixed methods designs to capture more nuanced insights regarding the influence of School Streets. A more comprehensive and robust evidence base would support the scalability of School Street interventions to promote active and independent mobility among children across Canada. Finally, creating car-free safe zones in public spaces can provide more opportunities for children to be outside the home, socialize with others, play, and become more familiar with their surroundings. This might also increase their independent mobility, which generally has a positive influence on their physical and mental health.

## Contributions to knowledge

What does this study add to existing knowledge?

- The implications of this study are multifaceted and significant for policymakers, urban planners, educators, and public health professionals alike. The findings underscore the potential effectiveness of School Street interventions in promoting active and independent modes of travel among elementary school children. This highlights the potential of School Streets as a promising strategy for enhancing children’s physical activity levels, reducing traffic congestion around schools, and creating more inclusive public spaces for children in urban contexts.
- Policymakers and urban planners could consider implementing School Street initiatives as part of broader efforts to create safer and more child-friendly environments around schools.

What are the key implications for public health interventions, practice, or policy?

- Policymakers and urban planners can consider implementing School Street initiatives (or similar non-permanent, low-cost urban interventions) as part of broader efforts to promote active transportation and create healthier communities.
- By prioritizing School Streets in urban planning and transportation policies, policymakers can contribute to reducing traffic-related injuries and increasing independent mobility and active transport use in children, thus supporting public health goals.

## Data Availability

Data will be made available on request.
